# Cholesterol Down-Regulates BK Channels Stably Expressed in HEK 293 Cells

**DOI:** 10.1371/journal.pone.0079952

**Published:** 2013-11-18

**Authors:** Wei Wu, Yan Wang, Xiu-Ling Deng, Hai-Ying Sun, Gui-Rong Li

**Affiliations:** 1 Department of Medicine, Li Ka Shing Faculty of Medicine, The University of Hong Kong, Pokfulam, Hong Kong, China; 2 Department of Physiology and Pathophysiology, School of Medicine, Xi'an Jiaotong University, Xi'an, China; 3 Department of Physiology, Li Ka Shing Faculty of Medicine, The University of Hong Kong, Pokfulam, Hong Kong, China; Rutgers-New Jersey Medical School, United States of America

## Abstract

Cholesterol is one of the major lipid components of the plasma membrane in mammalian cells and is involved in the regulation of a number of ion channels. The present study investigates how large conductance Ca^2+^-activated K^+^ (BK) channels are regulated by membrane cholesterol in BK-HEK 293 cells expressing both the α-subunit hKCa1.1 and the auxiliary β1-subunit or in hKCa1.1-HEK 293 cells expressing only the α-subunit hKCa1.1 using approaches of electrophysiology, molecular biology, and immunocytochemistry. Membrane cholesterol was depleted in these cells with methyl-β-cyclodextrin (MβCD), and enriched with cholesterol-saturated MβCD (MβCD-cholesterol) or low-density lipoprotein (LDL). We found that BK current density was decreased by cholesterol enrichment in BK-HEK 293 cells, with a reduced expression of KCa1.1 protein, but not the β1-subunit protein. This effect was fully countered by the proteasome inhibitor lactacystin or the lysosome function inhibitor bafilomycin A1. Interestingly, in hKCa1.1-HEK 293 cells, the current density was not affected by cholesterol enrichment, but directly decreased by MβCD, suggesting that the down-regulation of BK channels by cholesterol depends on the auxiliary β1-subunit. The reduced KCa1.1 channel protein expression was also observed in cultured human coronary artery smooth muscle cells with cholesterol enrichment using MβCD-cholesterol or LDL. These results demonstrate the novel information that cholesterol down-regulates BK channels by reducing KCa1.1 protein expression via increasing the channel protein degradation, and the effect is dependent on the auxiliary β1-subunit.

## Introduction

Large conductance Ca^2+^-activated and voltage-dependent K^+^ (BK, also called Maxi K) channels encoded by KCa1.1 (*KCNMA1* or Slo1) gene are widely distributed in smooth muscle, brain, pancreatic islets, etc., and are essential for the regulation of several key physiological processes including smooth muscle tone and neuronal excitability [1], [2]. BK channels are formed by a tetramer of pore-forming α-subunits (KCa1.1 or Slo1) with each α-subunit containing 7 transmembrane segments (S0–S6) and a large C-terminal cytoplasmic region [1]. The α-subunit KCa1.1 may interact with an auxiliary β-subunit [3], [4]; four types of β subunits are identified in different tissues [5], [6]. β1 is the predominant BK channel beta-subunit expressed in smooth muscle cells [7], [8] and regulates the channel sensitivity to Ca^2+^ ions [6], [9]. The expression of β1 subunit was found to be decreased in vascular smooth muscle cells in a rat hypertension model [10] and in patients with hypertension [11].

Activity of BK channels is up-regulated by different signal pathways including cAMP-dependent protein kinase A (PKA) and cGMP-dependent kinases [12]–[14], Src tyrosine kinases [15], insulin via MAPK activation [16]. In addition, the channel activity is found to be increased by epoxyeicosatrienoic acid [17] and arachidonic acid [18]. However, reports on the regulation of BK current by membrane cholesterol are controversial in different tissues/cells [19]–[23].

It has been reported that cholesterol enrichment suppresses BK channel activity in human melanoma IGR39 cells [20] and in reconstituted channels in lipid bilayers [21]. However, in rat uterine myocytes, cholesterol enrichment has no effect on BK current, while cholesterol depletion suppresses the current [24]. On the other hand, cholesterol depletion up-regulates the BK activity in colonic epithelial cells and vascular endothelial cells [25], [26]. The present study was designed to determine whether cholesterol-related alterations of BK channels, which are stably expressed in HEK 293 cells, are related to the channel protein level and/or β1 subunit using electrophysiology and molecular biology approaches. Our results demonstrated that the regulation of BK channels by cholesterol was dependent on the auxiliary β1 subunit.

## Materials and Methods

### Cell culture and gene transfection

Human KCa1.1 and KCa1.1-β1 pcDNA3.1 plasmids were generously provided by Dr. Christopher J. Lingle (Washington University, St. Louis, MO). The plasmids (4 µg) were transfected separately into HEK 293 cells (ATCC, Manassas, VA) in a 35 mm culture dish with Lipofectamine 2000 to establish stable HEK 293 cell lines. BK-HEK 293 cells stably express both hKCa1.1 (*KCNMA1*) and β1- (*KCNMB1*) subunits, and hKCa1.1-HEK 293 cells express only the *KCNMA1* gene. The cells were cultured in Dulbecco's modified Eagle's medium (DMEM, Invitrogen, Hong Kong, China) supplemented with 10% fetal bovine serum (FBS, Invitrogen) and 400 µg/ml G418 (Invitrogen). Cells used for electrophysiology were seeded on glass cover slips.

Human coronary artery smooth muscle cells were obtained from ScienCell Research Laboratory (Carlsbad, CA, USA), and cultured with α-MEM and F12 medium (Invitrogen) containing 15% FBS.

### Chemicals and solutions

Methyl-β-cyclodextrin (MβCD), cholesterol, and low density lipoprotein (LDL) were purchased from Sigma Chemical (St. Louis, MO). Lactacystin and bafilomycin A1 were purchased from Cayman Chemical (Ann Arbor, MI). Stock solutions were made with ethanol for lactacystin (20 mM) and bafilomycin A1 (10 mM). The stocks were divided into aliquots and stored at −20°C.

Tyrode's solution contained (mM) NaCl 140, KCl 5.4, MgCl_2_ 1.0, CaCl_2_ 1.8, 4-(2-hydroxyethyl)-1-piperazineethanesulfonic acid (HEPES) 10.0 and glucose 10 (pH adjusted to 7.3 with NaOH). For whole-cell recordings, the pipette solution contained (mM) KCl 20, K-aspartate 110, MgCl_2_ 1.0, HEPES 10, ethyleneglycoltetraacetic acid (EGTA) 5, GTP 0.1, Na_2_-phosphocreatine 5 and Mg-ATP 5 (pH adjusted to 7.2 with KOH). Free Ca^2+^ of 300 nM in the pipette solution was included as calculated using the CaBuf software created by Dr. G. Droogmans (Department of Physiology, KU Leuven, Leuven, Belgium).

### Cholesterol enrichment or depletion

The cholesterol enrichment or depletion was conducted in HEK 293 cell lines with cholesterol-saturated MβCD (MβCD-cholesterol) or MβCD with the ratio and the procedure as established previously [27], [28]. Briefly, a small volume of cholesterol stock solution made by dissolving cholesterol in chloroform: methanol (1∶1 vol/vol) was added to a glass tube and the solvent was evaporated, and then serum-free DMEM medium containing 5 mM MβCD was added to the dried cholesterol. The sealed tube was vortexed, sonicated, and incubated overnight in a shaking bath at 37°C. Cholesterol-saturated MβCD had a molar ratio of 1∶8 (cholesterol∶ MβCD).

In experiments with cholesterol enrichment or depletion, cells were washed three times with serum-free DMEM medium, and then incubated with the medium containing 5 mM MβCD-cholesterol or 5 mM MβCD for 2 h at 37°C and 5% CO_2_. After the treatment, the cells were washed three times with serum-free DMEM medium and incubated for at least 24 h in serum-free medium while maintaining the enriched or the depleted cholesterol. In the study of protein degradation, cells were treated or co-treated with bafilomycin A1 (1 µM) or lactacystin (20 µM) for 2 h, and ethanol (equivolume) was used as control. The cells were then used for electrophysiological recordings and/or molecular biological study.

### Determination of cellular cholesterol content

Cholesterol content in HEK 293 cells and human coronary artery smooth muscle cells was determined spectrophotometrically using a cholesterol/cholesteryl ester quantitation kit (Bio Vision, Mountain View, CA) following the manufacturer's instruction. Briefly, the detached cells were incubated for 10 min at room temperature in Tyrode's solution, and then homogenized in chloroform-Triton X-100. After addition of cholesterol probe, the cholesterol content was estimated in a colorimetric assay. The protein content of each sample was determined using a Bradford protein assay, and the cholesterol concentration was expressed as µg/mg of protein.

### Electrophysiology

BK channel current was recorded using a whole-cell patch configuration as described previously [29], [30]. Briefly, cells on a coverslip were transferred to a cell chamber (0.5 ml) mounted on the stage of an inverted microscope (Diaphot, Nikon, Japan) and superfused at ∼2 ml/min with Tyrode's solution. Borosilicate glass electrodes (1.2-mm OD) were pulled with a Brown-Flaming puller (model P-97, Sutter Instrument Co, Novato, CA) and had tip resistances of 2–3 MΩ when filled with the pipette solution. A 3-M KCl–agar bridge was used as the reference electrode. The tip potential was zeroed before patch pipette contact with the cell. After a giga-Ohm seal was obtained by applying a negative pressure, the cell membrane was ruptured by applying a gentle negative pressure to establish whole cell configuration. Series resistance was 3–6 MΩ and was compensated by 80% to minimize voltage errors. The liquid junction potential was not corrected in the experiment. Membrane currents were measured using an EPC-10 amplifier and Pulse software (Heka Elektronik GmbH, Lambrecht, Germany). Command pulses were generated by a 12-bit digital-to-analog converter controlled by Pulse software. Current signals were low-pass filtered at 5 kHz and stored in the hard disk of an IBM compatible computer. All experiments were conducted at room temperature (22–23°C).

### Immunocytochemistry

Immunostaining technique was employed to examine KCa1.1 expression in BK-HEK 293 cells with the procedure as described previously [30]. Briefly, the cells cultured on coverslips were washed with PBS, and fixed with PBS containing 2% paraformaldehyde (PFA) for 20 min, and subsequently permeabilized with PBS containing 0.1% Triton X-100 for 3 min. The cells were washed three times with PBS then incubated with blocking buffer (5% BSA in PBS) for 1 h. The cells were incubated at 4°C overnight with the primary antibody (anti-KCa1.1 antibody) diluted in PBS containing 3% BSA, washed with PBS, then incubated with the fluorescence-labeled secondary antibody (Invitrogen) in PBS with 3% BSA in the dark at room temperature for 1 h. The coverslip was then washed four times with PBS and mounted with ProLong Gold Anti-fade Reagent (Invitrogen, Hong Kong, China) for durable visualization. The coverslip was then observed and captured with confocal microscopy (Olympus FV300, Tokyo, Japan).

### Western blot analysis

The BK channel membrane protein was determined in BK-HEK 293 cells, hKCa1.1-HEK 293 cells, or human coronary artery smooth muscle cells with Western blot analysis with the procedure as described previously [30], [31]. Briefly, whole cell lysates were prepared with a modified RIPA buffer containing (mM) 50 Tris·HCl, 150 NaCl, 1 EDTA, 1 PMSF, 1 sodium orthovanadate, 1 NaF, 1 µg/ml aprotinin, 1 µg/ml leupeptin, 1 µg/ml pepstatin, 1% Nonidet P-40, 0.25% sodium deoxycholate, and 0.1% SDS. Protein concentration was determined using a Bio-Rad protein assay, and then used for Western blot analysis.

Cell lysates (40 µg) were mixed with sample buffer and denatured by heating to 95°C for 5 min. Samples were resolved via SDS-PAGE and transferred to nitrocellulose membranes. Membranes were blocked with 5% nonfat milk in Tris-buffered saline with Tween (TTBS) and then probed with primary antibodies at 4°C overnight with agitation. After wash with TTBS, the membranes were incubated with horseradish peroxidase-conjugated goat anti-rabbit or donkey anti-goat IgG antibody at 1∶5,000 dilution in TTBS at room temperature for 1 h. Membranes were washed again with TTBS and then processed onto X-ray film using an enhanced chemiluminescence detection system (GE Healthcare, Hong Kong, China). The membranes were then stripped to re-probe with an anti-GAPDH antibody. The relative intensity of Western blot was measured by a quantitative scanning densitometer and image analysis software (Bio-1D version 97.04).

### Statistical analysis

The data are expressed as mean±SEM. Nonlinear curve-fitting was performed using Sigmaplot (SPSS, Chicago, IL). Paired and/or unpaired Student's t-test were used as appropriate to evaluate the statistical significance of differences between two group means, and two-way ANOVA was used for multiple groups. Values of *P*<0.05 were considered to be statistically significant.

## Results

### BK channel current in HEK 293 cells

BK current showed a strong outward rectification in BK-HEK 293 cells stably expressing hKCa1.1 and the auxiliary β1 subunit. The current was reversibly inhibited by the BK channel blocker paxilline (1 µM) as shown in Figure S1 in File S1.

### Cholesterol and BK channel current

The effect of MβCD, MβCD-cholesterol complex or LDL on membrane cholesterol content was determined in BK-HEK 293 cells. MβCD (5 mM) significantly reduced the cellular cholesterol content from 13.0±1.9 µg/mg in control to 7.4±1.0 µg/mg protein (n = 5, *P*<0.05 vs. control), whereas 5 mM MβCD-cholesterol complex increased the cholesterol content to 19.8±2.3 µg/mg protein (n = 5, *P*<0.05 vs. control). LDL increased the cholesterol content to 21.5±4.2 µg/mg protein (n = 5, *P*<0.05 vs. control), while the increase was reduced to 14.4±1.6 in cells with both LDL and MβCD (n = 5, *P*<0.05 vs. LDL alone).

**Figure 1:**
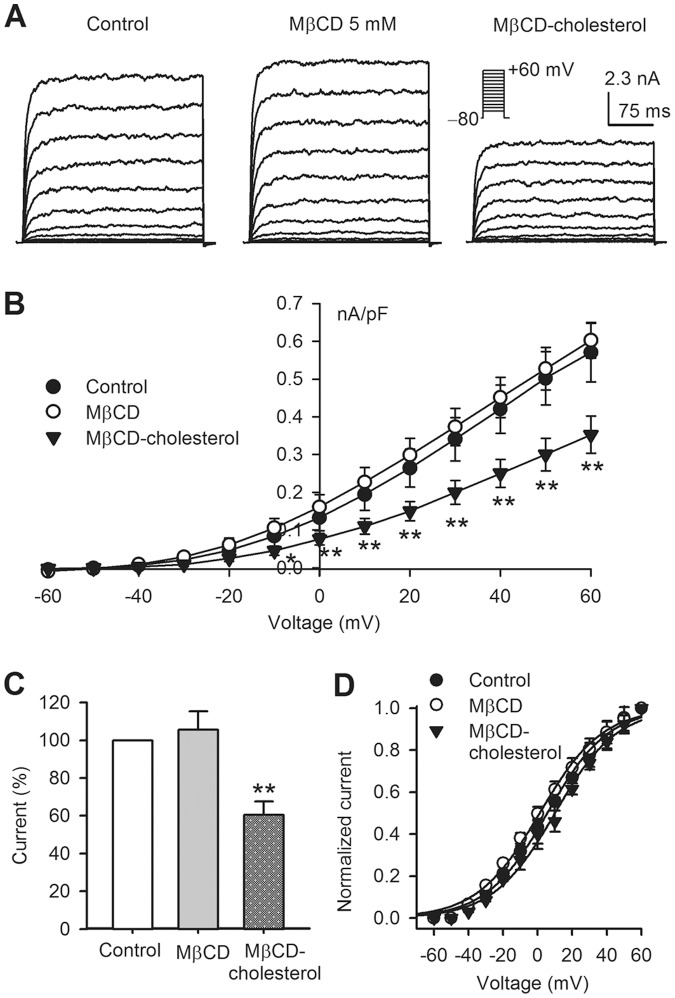
Effects of cholesterol on BK channel current. **A**. Voltage-dependent BK current elicited with 300-ms voltage steps between −70 and +60 mV from a holding potential of -80 mV in BK-HEK 293 cells without treatment (control) or with cholesterol depletion (MβCD) or cholesterol enrichment (MβCD-cholesterol). The current was recorded with the pipette solution containing 300 nM free Ca^2+^. **B**. *I-V* relationships of BK current in control (n = 25), with cholesterol depletion or cholesterol enrichment (n = 25, *P<0.05 or **P<0.01 vs. control). **C**. BK current (as a percentage of control) at +60 mV in control (n = 25), cholesterol depletion (n = 23, *P* = NS vs. control), and cholesterol enrichment (n = 33, **P<0.01 vs. control). **D**. Activation curves calculated with *I-V* relationships of the current in panel **B** and fitted to the Boltzmann function: y = 1/{1+exp[(Vm-V_1/2_)/S]}, where V_m_ is membrane test potential, V_1/2_ is the midpoint of activation potential, and S is slope.

Figure 1 illustrates the effect of cholesterol depletion or enrichment on BK channel current. Depletion of membrane cholesterol with 5 mM MβCD slightly increased BK current, while cholesterol enrichment with MβCD-cholesterol complex remarkably reduced the current amplitude (Figure 1A). The *I-V* relationships (Figure 1B) of BK current showed a reduced current density at test potentials between −10 and +60 mV in cells treated with cholesterol enrichment (n = 25, *P*<0.05 or *P*<0.01 vs. control) and a slight increase in the current density in cells with cholesterol depletion. At +60 mV, the current was increased to 105.6±9.7% of control in cells with cholesterol depletion (n = 23, *P* = NS vs. control), and reduced to 60.5±7.2% of control in cells with cholesterol enrichment (n = 33, *P*<0.01 vs. control) (Figure 1C).

The mean values of the variables of the voltage-dependent activation calculated from the *I-V* relationships (Figure 1B) of BK current for individual cells based on the formulation g = I/(V_t_-V_r_) as described previously [32], where I is the current at the test voltage (V_t_), and V_r_ is the measured reversal potential (−70 mV). The voltage-dependence of the conductance normalized to the conductance at +60 mV is shown in Figure 1D. The variables were fitted to a Boltzmann function to obtain the half voltage (V_1/2_) of BK channel activation. The V_1/2_ of BK channel activation was 5.5±1.2 mV in control, 1.4±1.1 mV in MβCD, 8.1±1.6 mV in MβCD-cholesterol (n = 16 for each group, *P* = NS vs. control). No significant difference in slope factor of the curves was observed with different treatments. These results suggest that cholesterol enrichment significantly decreases BK current density without affecting the voltage-dependent activation in BK-HEK 293 cells.

### Single channel activity of BK channels in cells with cholesterol-enrichment

**Figure 2:**
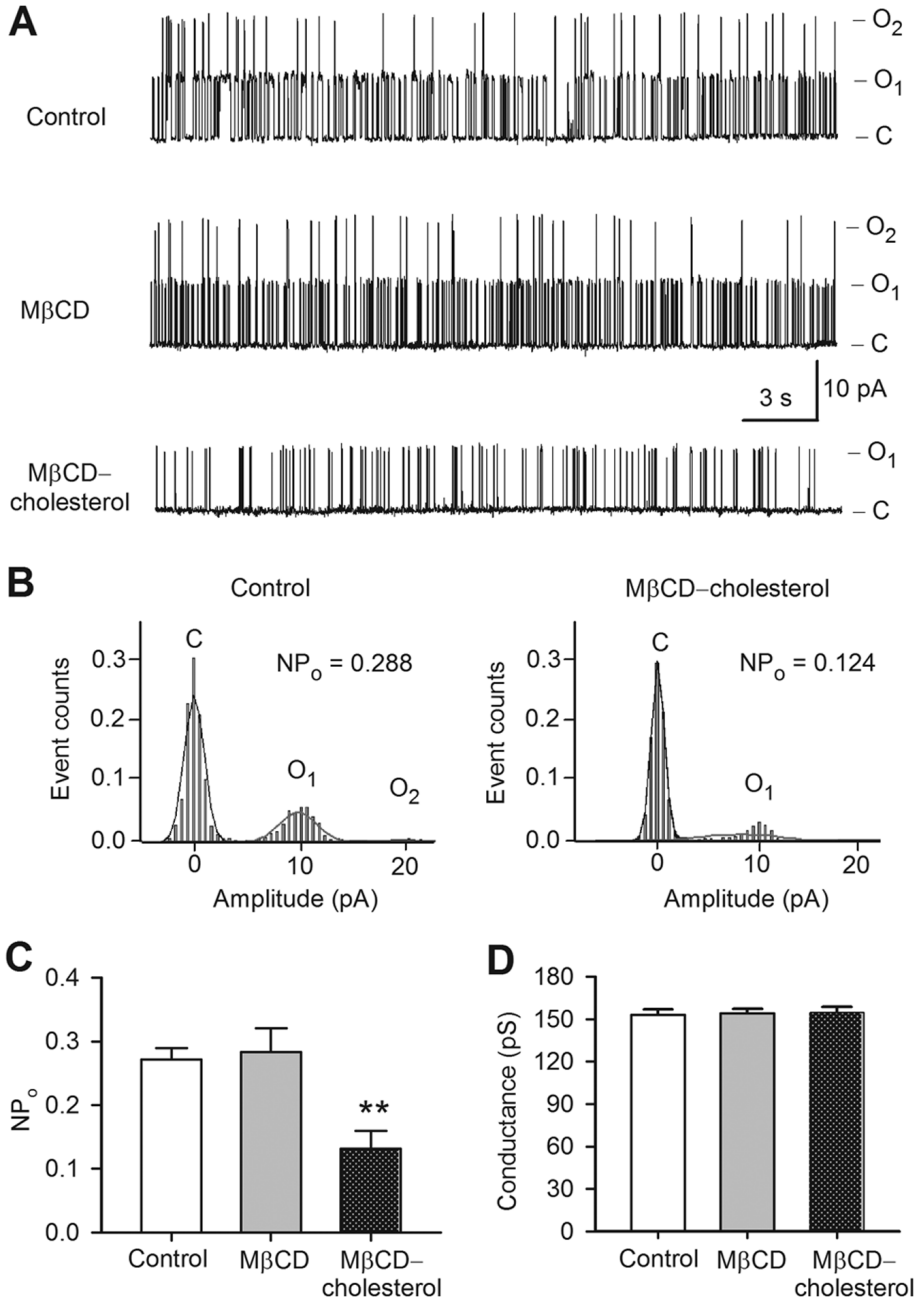
Single channel activity of BK channels. **A**. Single channel activity of BK channels recorded with cell-attached mode at +60 mV in cells treated with 5 mM MβCD or MβCD-cholesterol. The labels C, O_1_ and O_2_ indicate the close level, 1^st^ and 2^nd^ opening levels of the channel. **B**. Histograms of open probability of BK single channels in cells treated without (control) or with cholesterol enrichment (MβCD-cholesterol). **C**. Mean values of open probability of BK channels in control (n = 7), MβCD (n = 6), and MβCD-cholesterol (n = 7, **P<0.01 vs. control). **D**. Conductance of BK channels in control, MβCD, and MβCD-cholesterol (n = 6–7, *P* = NS vs. control).

To determine whether the reduced current of BK channels is related to the alteration in single channel open probability or unitary conductance, single channel activity of BK channels was recorded in cell-attached mode with the procedure as described previously [33] in cells treated with MβCD or MβCD-cholesterol. Figure 2A illustrates the raw data of single channel activity of BK channels. Two open levels of single channel were observed in control cells and in MβCD-treated cells, but not in cells treated with MβCD-cholesterol. In addition, single channel opening events were clearly reduced in cells with cholesterol enrichment (Figure 2A and 2B), suggesting that both current density and open probability are reduced. The analysis of single channel open probability revealed that the mean open probability of single BK channel (Figure 2 C) was significantly lower in cells with cholesterol enrichment (n = 7, *P*<0.01 vs. control). However, unitary conductance of single channels was not affected in cells with cholesterol depletion or cholesterol enrichment (Figure 2D). These results suggest that the decreased BK current by cholesterol-enrichment is likely related to the reduction of channel density and channel open probability.

### Cholesterol enrichment and KCa1.1 protein expression

**Figure 3:**
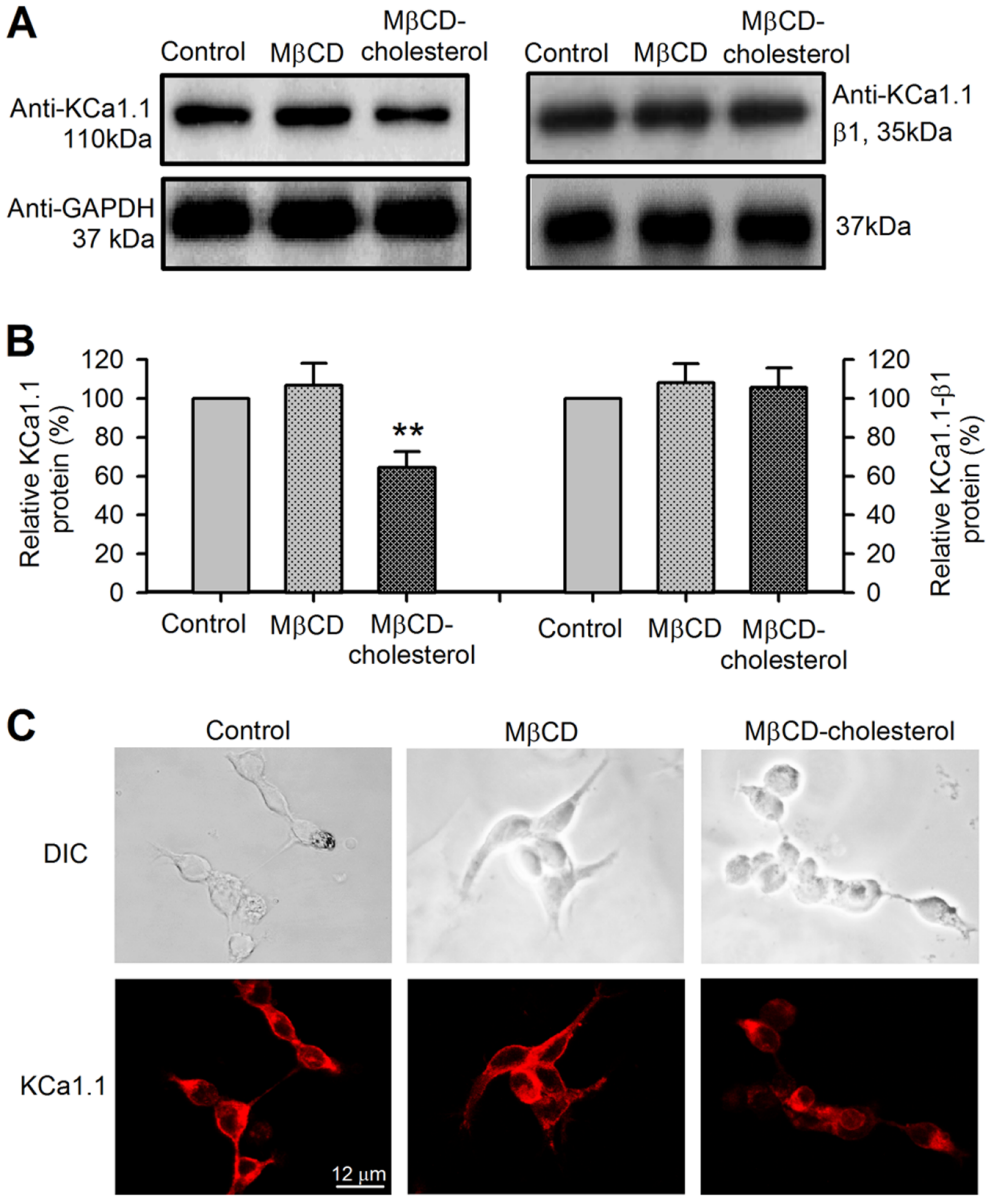
Protein expression of BK channels in BK-HEK 293 cells with co-expressing auxiliary β1-subunit. **A**. Western immunoblots showing KCa1.1 protein and the auxiliary β1-subunit protein in cells treated with cholesterol depletion (MβCD) or cholesterol enrichment (MβCD-cholesterol). **B**. Mean percentage values of KCa1.1 protein and the β1-subunit protein in cells with different treatments (n = 6–9, **P<0.01 vs. control). **C**. Immunocytochemistry showing membrane expression of KCa1.1 protein in cells treated with cholesterol depletion (MβCD) or cholesterol enrichment (MβCD-cholesterol). Cholesterol enrichment induced a reduction of membrane expression of BK channels.

To examine whether the reduced BK channel current density is related to the decrease of KCa1.1 protein level, KCa1.1 protein and β1-subunit protein were determined using Western blot analysis in cells with different treatments. Figure 3 illustrates the Western immunoblots and mean percentage values of KCa1.1 protein and β1-subunit protein, and immunocytochemical staining in cells treated with MβCD or MβCD-cholesterol. The KCa1.1 protein level was reduced by MβCD-cholesterol. Cholesterol enrichment with MβCD-cholesterol reduced the expression of KCa1.1 protein to 64.4±8.1% (Figure 3B) of control (n = 9, *P*<0.01 vs. control). However, KCa1.1 β1-subunit protein (Figure 3B) was not decreased by MβCD-cholesterol (n = 8, *P* = NS vs. control). The results (Figure 3C) of the immunocytochemistry also showed a reduced KCa1.1 protein on the cell membrane in cells with cholesterol-enrichment. These results indicate that decreased KCa1.1 protein expression is also involved in the reduced BK current by cholesterol-enrichment.

### Effect of low density lipoprotein on BK channels

**Figure 4:**
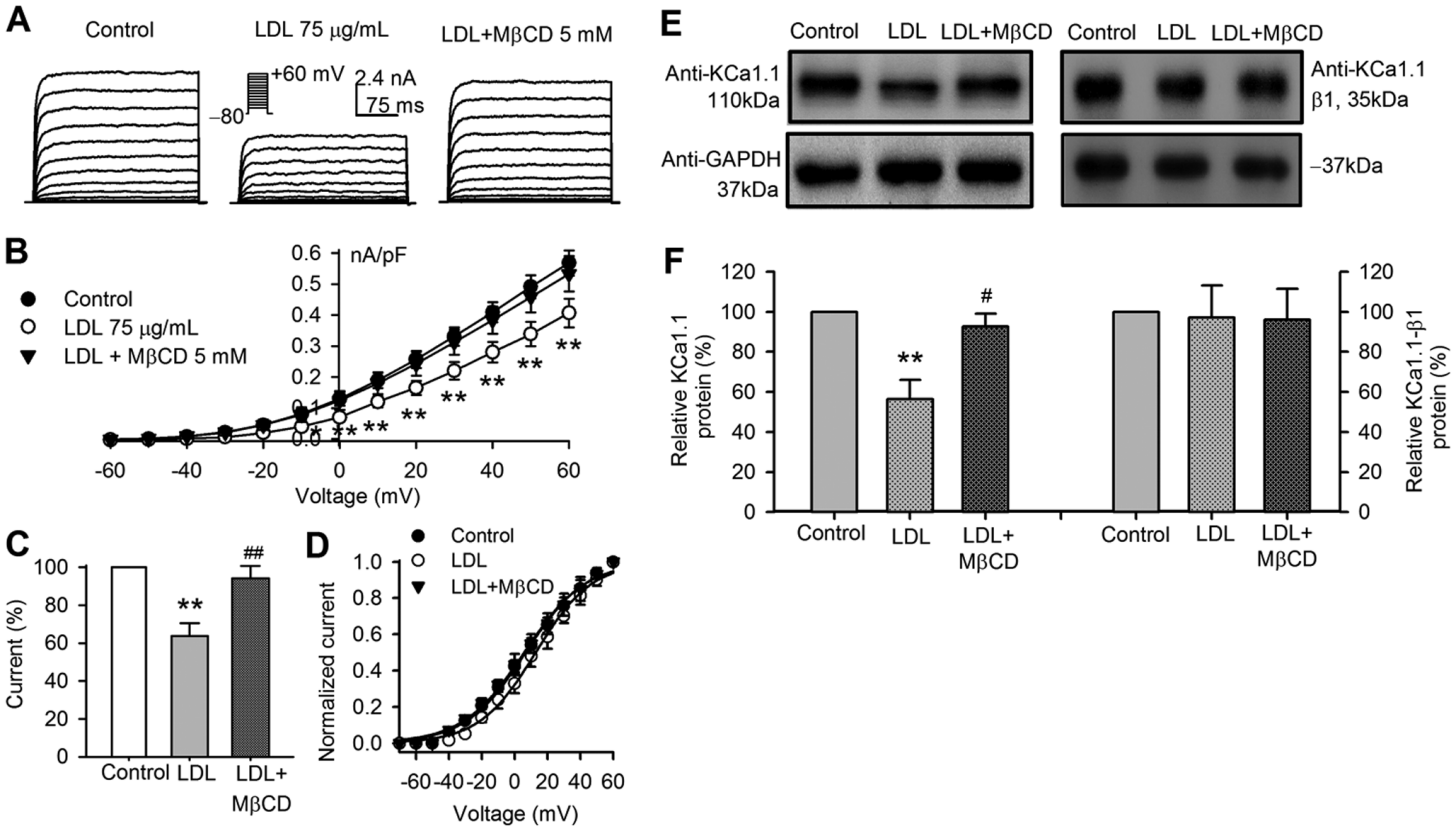
Effects of LDL on BK channel current in BK-HEK 293 cells. **A**. Voltage-dependent BK current recorded with voltage protocol as shown in the inset in cells treated with LDL (75 µg/mL) or LDL plus MβCD treatment. **B**. *I-V* relationships of BK current in control, LDL or LDL plus MβCD treatment (n = 25, *P<0.05; **P<0.01 vs. control). **C**. Percentage values compared to control of BK current at +60 mV in control (n = 25), with LDL (n = 32, **P<0.01 vs. control), and LDL plus MβCD treatment (n = 10, ^##^ *P*<0.01 vs. LDL alone). **D**. Activation variables calculated with *I-V* relationships of the channel current in panel **B** were fitted to a Boltzmann function. **E**. Western immunoblots showing KCa1.1 protein and the auxiliary β1-subunit protein in cells treated with LDL or LDL plus MβCD. **F**. Mean percentage values of KCa1.1 protein and the auxiliary β1-subunit protein in cells with LDL or LDL plus MβCD (n = 6-9, **P<0.01 vs. control, ^#^ *P*<0.05 vs. LDL alone).

The effect of the cholesterol donor low density lipoprotein (LDL) on BK channels was determined in BK-HEK 293 cells incubated with LDL (75 µg/mL) for 48 h. Figure 4A shows that BK current was decreased in cells treated with LDL, and the effect was reduced in cells treated with LDL plus 5 mM MβCD. The *I-V* relationships of BK current in Figure 4B show that LDL decreased the current density at −10 to +60 mV (n = 25, *P*<0.05 or *P*<0.01 vs. control). The current at +60 mV was reduced to 63.7±6.8% of control (n = 32, *P*<0.01 vs. control) in cells treated with LDL, and reversed to 94.2±6.5% of control (n = 10, *P*<0.01 vs. LDL alone) in cells treated with both LDL and MβCD (Figure 4C). The variables of voltage-dependent activation of BK channels calculated from the *I-V* relationships (Figure 4B) were fitted to a Boltzmann function (Figure 4D), and the V_1/2_ of the channel activation was 6.6±1.4 mV in control, 10.2±1.7 mV in LDL, and 5.8±1.5 mV in LDL plus MβCD (n = 10 for each group, *P* = NS vs. control), suggesting that LDL, like MβCD-cholesterol, reduces BK current without significantly affecting the voltage-dependent activation.

The results (Figure 4E and 4F) from Western blot analysis demonstrated that LDL, similar to MβCD-cholesterol, reduced KCa1.1 protein (by 44.7±9.8%, n = 6, *P*<0.01 vs. control), but not β1-subunit protein. The reduced KCa1.1 protein was reversed in cells treated with LDL plus MβCD. These results indicate that LDL, like MβCD-cholesterol, reduces BK current density and membrane channel protein.

### Cholesterol enrichment and KCa1.1 channel protein degradation

**Figure 5:**
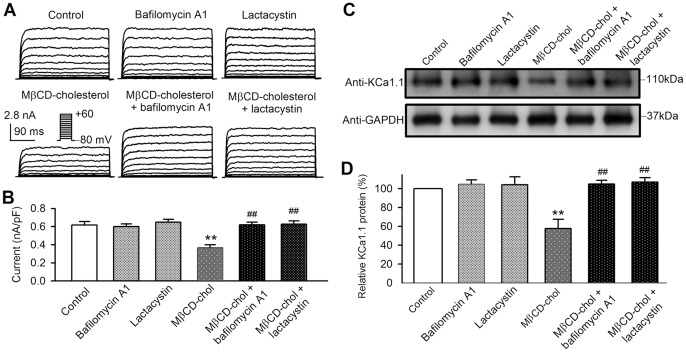
Cholesterol regulation and channel protein degradation in BK-HEK 293 cells with co-expressing β1-subunit. **A**. BK channel current recorded with the voltage protocol (inset) in cells treated with bafilomycin A1, lactacystin, MβCD-cholesterol, MβCD-cholesterol plus bafilomycin A1, or MβCD-cholesterol plus lactacystin. **B**. BK channel current at +60 mV in cells treated with bafilomycin A1 (n = 16, *P* = NS vs. control), lactacystin (n = 15, *P* = NS vs. control), MβCD-cholesterol (MβCD-chol, n = 19, **P<0.01 vs. control), MβCD-cholesterol plus bafilomycin A1 (n = 14, ^##^ *P*<0.01 vs. MβCD-cholesterol), or MβCD-cholesterol plus lactacystin (n = 18, ^##^ *P*<0.01 vs. MβCD-cholesterol). **C**. Western immunoblots showing KCa1.1 protein in cells treated with bafilomycin A1, lactacystin, MβCD-cholesterol, MβCD-cholesterol plus bafilomycin A1, or MβCD-cholesterol plus lactacystin. **D**. Mean percentage values of KCa1.1 protein in cells treated with bafilomycin A1, lactacystin, MβCD-cholesterol, MβCD-cholesterol plus bafilomycin A1, or MβCD-cholesterol plus lactacystin (n = 6, **P<0.01 vs. control; ^##^ *P*<0.01 vs. MβCD-cholesterol).

It is generally recognized that protein degradation is mainly mediated by proteasome pathway and lysosome pathway [34]. To determine whether the channel degradation process is involved in the reduction of KCa1.1 protein expression by cholesterol enrichment, the classical proteasome inhibitor lactacystin [35] and the lysosomal function inhibitor bafilomycin A1 [36] were applied in BK-HEK 293 cells. Bafilomycin A1 (1 µM) or lactacystin (20 µM) had no effect on BK channel current. Interestingly, both inhibitors reversed the BK current reduction induced by MβCD-cholesterol (Figure 5A). The mean values of current density (Figure 5B) at +60 mV were not affected by bafilomycin A1 (n = 16, *P* = NS vs. control) or lactacystin (n = 18, *P* = NS vs. control), but reduced by MβCD-cholesterol (n = 15, *P*<0.01 vs. control), and the reduction by MβCD-cholesterol was reversed by the addition of bafilomycin A1 (n = 18, *P*<0.01 vs. MβCD-cholesterol) or lactacystin (n = 16, *P*<0.01 vs. MβCD-cholesterol). Figure 5C and 5D illustrate the Western immunoblots and mean percentage values of KCa1.1 protein level in cells treated with bafilomycin A1, lactacystin, MβCD-cholesterol, MβCD-cholesterol plus bafilomycin A1, or MβCD-cholesterol plus lactacystin. The KCa1.1 protein was reduced by cholesterol enrichment with MβCD-cholesterol, and the effect was reversed by the lysosomal inhibition with bafilomycin A1 or the proteasome inhibition with lactacystin. These results indicate that the reduction of BK channels by cholesterol enrichment is related to facilitating channel protein degradation.

### β1 subunit-dependent regulation of BK channels by cholesterol

**Figure 6:**
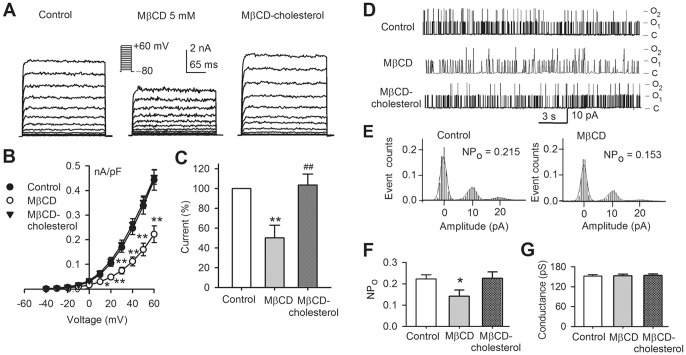
Cholesterol effect on KCa1.1 current. **A**. Voltage-dependent KCa1.1 current recorded in hKCa1.1-HEK 293 cells with the voltage protocol (*inset*) in cells treated with cholesterol depletion (MβCD) or cholesterol enrichment (MβCD-cholesterol). **B**. *I-V* relationships of hKCa1.1 current in control (n = 19), cholesterol depletion (n = 16, *P<0.05 or **P<0.01 vs. control at 0 to +60 mV) or cholesterol-enrichment (n = 11, *P* = NS vs. control). **C**. Percentage values of hKCa1.1 current at +60 mV in control (n = 16), cholesterol depletion (n = 19, **P<0.01 vs. control) or cholesterol enrichment (n = 11, ^##^ *P*<0.01 vs. MβCD alone). **D**. Single channel activity of hKCa1.1 channels recorded in cell-attached mode at +60 mV in cells treated with 5 mM MβCD or MβCD-cholesterol. The labels C, O_1_ and O_2_ indicate the close level, 1^st^ and 2^nd^ opening levels of the channel. **E**. Histograms of open probability of hKCa1.1 single channels in cells treated without (control) or with cholesterol depletion (MβCD). **F**. Mean values of open probability of hKCa1.1 channels in control (n = 8), MβCD (n = 6), and MβCD-cholesterol (n = 6, *P<0.05 vs. control). **G**. Conductance of hKCa1.1 channels in control, MβCD, and MβCD-cholesterol (n = 6–8, *P* = NS vs. control).

To determine whether the auxiliary β1 subunit of BK channels is critical for the cholesterol regulation, further experiments were performed in hKCa1.1-HEK 293 cells not co-expressing the β1-subunit. Figure 6A shows that voltage-dependent current traces recorded in representative cells treated with MβCD or MβCD-cholesterol. It is interesting to note that the current amplitude of KCa1.1 channels is reduced in the cells treated with MβCD, and slightly increased in the cells incubated with MβCD-cholesterol. Figure 6B shows the *I-V* relationships of KCa1.1 current with a relatively positive threshold potential of the channel activation, which implies the reduced Ca^2+^-sensitivity. The current density was reduced at test potentials between +10 and +60 mV in cells with cholesterol depletion using 5 mM MβCD (n = 19, *P*<0.05 or *P*<0.01 vs. control). The current at +60 mV was reduced to 52.3±12.8% of control (n = 19, *P*<0.01 vs. control). No effect was observed in cells with cholesterol enrichment (n = 11, *P* = NS vs. control) (Figure 6C).

To determine whether MβCD-induced reduction of KCa1.1 current is related to the alteration in single channel open probability or unitary conductance, single channel activity of KCa1.1 channels was recorded in hKCa1.1-HEK 293 cells treated with MβCD or MβCD-cholesterol. Figure 6D illustrates the single channel activity of KCa1.1 channels. Two open levels of single channel were observed in control cells, MβCD cells, and in MβCD-cholesterol cells, suggesting that channel density would not be altered. However, opening events were reduced in MβCD cells. The analysis of single channel open probability (Figure 6E) revealed that the mean open probability of single KCa1.1 channel (Figure 6F) was significantly decreased in cells treated with MβCD (n = 5, *P*<0.05 vs. control). However, unitary conductance of single channels was not affected in cells with cholesterol depletion or cholesterol enrichment (Figure 6G). These results suggest that the decreased KCa1.1 current by MβCD is likely related to the reduction of channel open probability in hKCa1.1-HEK 293 cells.

It is interesting to note that KCa1.1 protein, as shown in Figure S2 in File S1, was not affected by cholesterol depletion or cholesterol enrichment in hKCa1.1-HEK 293 cells, which differs from that in BK-HEK 293 cells expressing both hKCa1.1 and β1-subunits. The decrease of hKCa1.1 current by cholesterol depletion with MβCD was not related to reduction of the channel protein, suggesting that the auxiliary β1-subunit of BK channels plays a crucial role in the channel regulation by cholesterol, and MβCD shows a direct inhibition of the channel by reducing the open probability in hKCa1.1-HEK 293 cells.

### Cholesterol enrichment and KCa1.1 expression in human coronary artery smooth muscle cells

To investigate whether cholesterol enrichment affects native KCa1.1 expression, the effect of MβCD, MβCD-cholesterol complex, or LDL on membrane cholesterol content was determined in cultured human coronary artery smooth muscle cells that express the auxiliary β1-subunit [37]. MβCD (5 mM) significantly reduced the cellular cholesterol content from 7.7±1.1 µg/mg in control to 3.9±0.8 µg/mg protein (n = 5, *P*<0.05 vs. control), whereas 5 mM MβCD-cholesterol complex increased the cholesterol content to 12.0±1.7 µg/mg protein (n = 5, *P*<0.05 vs. control. LDL increased the cholesterol content to 12.8±1.2 µg/mg protein (n = 5, *P*<0.01 vs. control), while the increase was reduced to 5.6±1.2 µg/mg in cells co-treated with both LDL and MβCD (n = 5, *P*<0.05 vs. LDL alone). It should be noted that cholesterol content is lower in cultured human coronary artery smooth muscle cells than that in HEK 293 cells (7.7 µg/mg vs. 13 µg/mg), suggesting that the basal content of membrane cholesterol is cell type-dependent.

**Figure 7:**
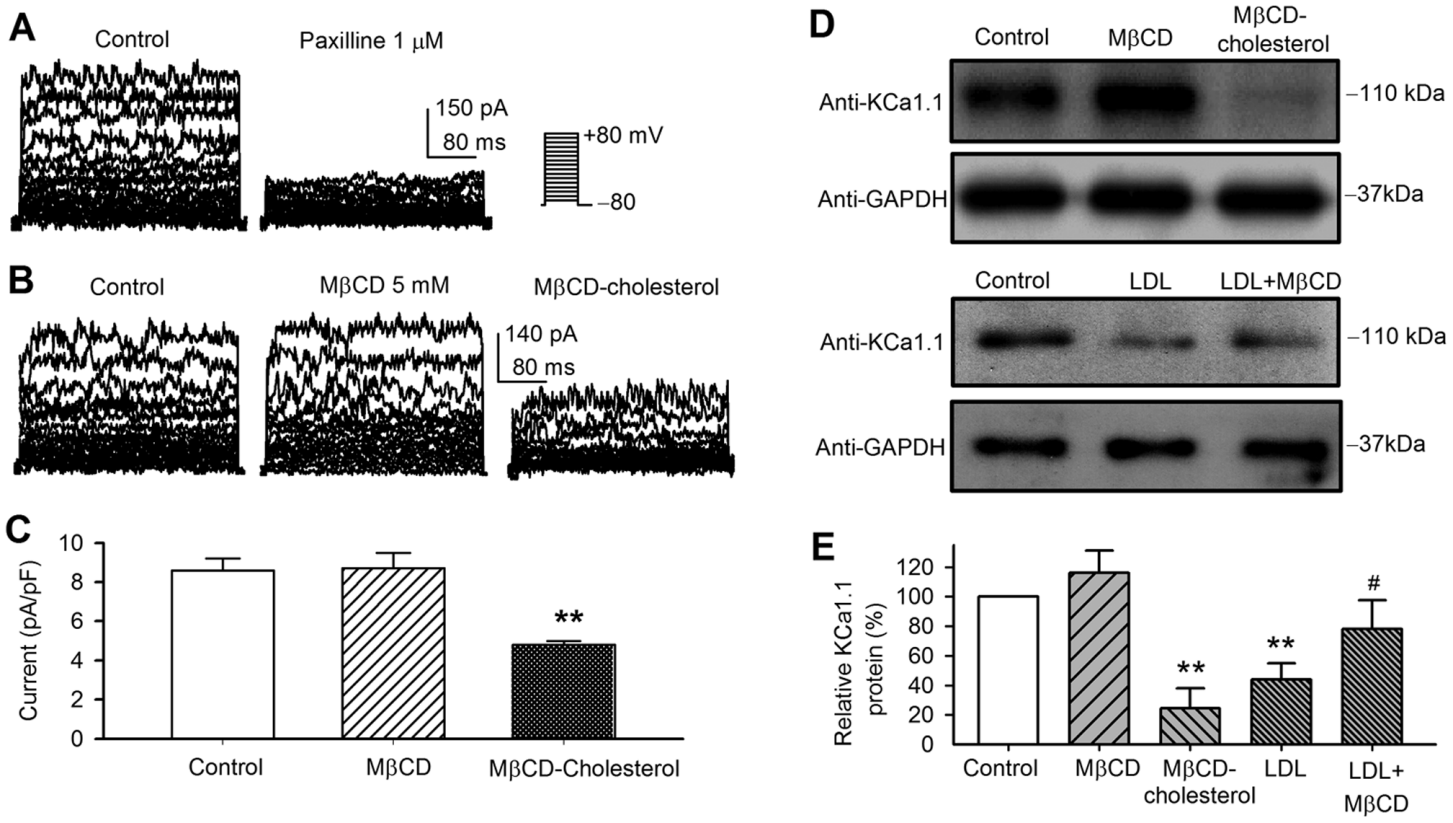
Effect of cholesterol on KCa1.1 protein in cultured human coronary artery smooth muscle cells. A. BK current was recorded in a representative human coronary artery smooth muscle cell with the voltage protocol shown in the inset, and the current was inhibited by 1 µM paxilline. B. BK current was recorded in cells treated without/with MβCD or MβCD-cholesterol. C. Mean values of BK current at +80 mV in cells treated with MβCD (n = 5) or MβCD-cholesterol (n = 5, **P<0.01 vs. control, n = 6). D. Western immunoblots showing KCa1.1 protein in human coronary artery smooth muscle cells treated with cholesterol depletion (MβCD), cholesterol enrichment (MβCD-cholesterol), LDL, or LDL plus MβCD. E. Mean percentage values of BK protein in cells with different treatments (*n* = 4–6, **P<0.01 vs. control, ^#^ *P*<0.05 vs. LDL alone).

BK current was recorded in cultured human coronary artery smooth muscle cells treated with MβCD, MβCD-cholesterol complex or LDL. We determined the whole-cell BK current in cells without/with treatment of MβCD or MβCD-cholesterol complex (Figure 7A–7C); we were unable to record the current in cells treated with LDL or LDL plus MβCD due to difficulty in obtaining a tight-seal in these cells for the whole-cell recording. BK current was inhibited by paxilline in human coronary artery smooth muscle cells (Figure 7A). The current was reduced in cells treated with MβCD-cholesterol complex, but not in cells treated with MβCD (Figure 7B). BK current at +80 mV (Figure 7C) was decreased to 56% of control (n = 5, *P*<0.01 vs. control) in cells treated with MβCD-cholesterol complex.

The KCa1.1 protein expression level was then determined in cultured human coronary artery smooth muscle cells with different treatments. Figure 7D shows the Western blots in cultured human coronary artery smooth muscle cells. KCa1.1 protein was also decreased by cholesterol enrichment with MβCD-cholesterol or LDL in human coronary artery smooth muscle cells. The KCa1.1 protein level (Figure 7E) was decreased to 24.6±13.3% of control in cells treated with MβCD-cholesterol (n = 9, *P*<0.01 vs. control), and 44.1±10.9% in cells treated with LDL (n = 6, *P*<0.01 vs. control). Cholesterol depletion with MβCD slightly increased KCa1.1 protein (116.1±15.1%, n = 9, *P* = NS vs. control). LDL-induced reduction of KCa1.1 protein was significantly reversed by co-incubation with 5 mM MβCD (78.1±19%, n = 6, *P*<0.05 vs. LDL alone). These results indicate that KCa1.1 channels in human coronary artery smooth muscle cells are down-regulated by cholesterol-enrichment with MβCD-cholesterol or LDL, similar to KCa1.1 expressed in BK-HEK 293 cells.

## Discussion

The present study has demonstrated that the cholesterol enrichment reduces the current density, single channel open probability and KCa1.1 protein expression level in BK-HEK 293 cells. The reduction of KCa1.1 protein by cholesterol enrichment can be reversed by proteasome inhibition with lactacystin or the lysosome inhibition with bafilomycin A1, suggesting that cholesterol enrichment-induced BK channel reduction involved protein degradation. However, cholesterol enrichment did not induce BK channel suppression in hKCa1.1-HEK 293 cells that do not express the β1-subunit. Therefore, the regulation of BK channels by cholesterol depends on the auxiliary β1-subunit. The observation of the β1-subunit dependent regulation of BK channels by cholesterol is supported by a recent report [38].

Several ion channels are regulated by the alterations of membrane cholesterol [39]. Usually, increased membrane cholesterol reduces channel activity, as seen in Kir2.x [40], [41], Kir6.x [42], voltage-gated Na^+^ channels [43], Ca^2+^ channels [44], [45]. On the other hand, several types of ion channels, such as Kir4.x [46] are inhibited by cholesterol depletion. Cholesterol modifies kinetic properties and current-voltage dependence of Kv1.5 and Kv2.1 channels [47]. Depletion of membrane cholesterol results in a positive shift of the activation potential and acceleration of the deactivation rate of hERG channels [48]. A recent study has reported that hERG and KCNQ channels are decreased by cholesterol enrichment via a reduced activity of phosphatidylinositol 4, 5-bisphosphate (PIP2) [49].

Cholesterol regulation of BK channels is complicated. Both down-regulation and up-regulation have been reported in various tissue types [39]. The up-regulation of BK current by reducing membrane cholesterol was observed in human endothelial cells [26], myometrial smooth muscle cells [50], melanoma IGR39 cells [20], and mouse colonic epithelial cells [25]. Nonetheless, down-regulation of the channel by lowering cholesterol level is reported in rat uterine myocytes [24] and human glioma cells [51]. The present study provides the novel information that the regulation of BK channels by cholesterol is dependent on the auxiliary β1-subunit. In hKCa1.1-HEK 293 cells without β1-subunit expression, cholesterol enrichment resulted in no significant alteration in current density or channel protein, while cholesterol depletion using MβCD resulted in a reduced current but no decrease in channel protein.

In BK-HEK 293 cells expressing both KCa1.1 and β1-subunits and in human coronary smooth muscle cells, cholesterol enrichment reduced current density and channel protein level. A previous study reported that hypercholesterolemia decreased the expression of β1-subunit of BK channels in cells of Oddi sphincter from rabbits on a high cholesterol diet [52]. However, we found that β1-subunit protein of BK channels was not affected by cholesterol enrichment or cholesterol depletion in BK-HEK 293 cells. This may suggest that the regulation of the β1-subunit by cholesterol is likely dependent on species and/or tissue type.

It has been suggested that cholesterol may regulate ion channels by hydrophobic mismatch between the transmembrane domains and the lipid bilayer [53]. The ion channels are associated with cholesterol-rich membrane domains lipid rafts (10–200 nm) which are “heterogeneous, highly dynamic, sterol and sphingolipid enriched domains that compartmentalize cellular processes” [54]. Ion channels may go through a change in conformation within the viscous medium of the lipid membrane that may cause deformation of the lipid bilayer surrounding the channel [39]. Kir2.x channel regulation by cholesterol is an example of the cholesterol interaction with lipid rafts and the channel protein [55], [56].

So far, there is no confirmed model that accounts for the diversity of cholesterol effects on BK channels. Cholesterol-dependent modulation of the channel activity in native cellular membranes has been found to be related to its localization to lipid rafts [39]. The modulation of BK channels by cholesterol is believed to be mainly due to the changes in biophysical properties of the lipid bilayer [21]. It should be noted that the human BK channel α-subunit KCa1.1 expressed in HEK 293 cells alone or in combination with any of the four known β-subunits was insensitive to acute application of cholesterol (10 µM) [57]. However, expression of BK channels cloned from rat cerebral artery myocytes reconstituted in binary phospholipid bilayers was inhibited by cholesterol in a β1-subunit independent manner [58].

Cholesterol depletion or interference with caveolin scaffolding decreased raft association with the channels, and upregulated BK current via destabilization of lipid rafts [24], [50], [51]. However, Weaver and colleagues demonstrated that cholesterol depletion inhibited the intricate functional association of BK channels with IP3 receptors thus resulted in a strong reduction of the channel current [51].

The present study found that the cholesterol regulation of BK channels was β1-subunit dependent. In hKCa1.1-HEK 293 cells without β1-subunit expression, cholesterol depletion with MβCD reduced the current density without reduction in KCa1.1 protein expression. However, in BK-HEK 293 cells expressing both KCa1.1 and auxiliary β1-subunits, cholesterol enrichment decreased the current density and KCa1.1 protein expression. The decreased KCa1.1 protein expression by cholesterol enrichment was also observed in cultured human coronary artery smooth muscle cells. These results support the notion that the auxiliary β1-subunit of BK channels plays a critical role in the regulation of the channel by cholesterol.

In the present study, we found that cholesterol decreased the open probability of BK channels, consistent with the previous reports (see review, [59]). It has been proposed that BK channels are regulated by cholesterol via insertion into the cell membrane and alteration of the bilayer physical properties, thereby leading to a direct recognition of the sterol by a protein surfacein the BK channel [59]. The alteration of both the protein conformation and physical properties of the lipid that surround the channel membrane-spanning regions by cholesterol [59] likely correlates with the present finding that the facilitation of channel protein degradation is involved in the cholesterol enrichment-induced reduction of BK channels, and the effect is dependent on the β1-subunit. It is interesting to note that the β1-subunit protein is not affected by the degradation system in cells with cholesterol enrichment.

Collectively, the present study has demonstrated that BK channels are down-regulated by membrane cholesterol-enrichment via increasing the channel protein degradation, and the effect is dependent on the auxiliary β1-subunit. This may provide an explanation for the controversial results obtained from different tissues/cells. The tissue-specific expression of the auxiliary β1-subunit may account for the different response to cholesterol.

## Supporting Information

File S1 **Supporting figures.** Figure S1. Membrane current in a HEK 293 cell stably expressing hKCa1.1 (*KCNMA1*) and the auxiliary β1 subunit (*KCNMB1*) is inhibited by paxilline. Figure S2. Cholesterol has no effect on KCa1.1 protein expression in hKCa1.1-HEK 293 cells without expressing auxiliary β1-subunits.
(PDF)
