# Supplementary material for: Cholesterol Down-Regulates BK Channels Stably Expressed in HEK 293 Cells
Source: PLoS One. 2013 Nov 18;8(11):e79952. doi: 10.1371/journal.pone.0079952 (PMC3832390; doi:10.1371/journal.pone.0079952)
Supplement: File S1 — Supporting figures. Figure S1. Membrane current in a HEK 293 cell stably expressing hKCa1.1 (KCNMA1) and the auxiliary β1 subunit (KCNMB1) is inhibited by paxilline. Figure S2. Cholesterol has no effect on KCa1.1 protein expression in hKCa1.1-HEK 293 cells without expressing auxiliary β1-subunits. (PDF) [file pone.0079952.s001.pdf]

Supplementary data

Figure S1

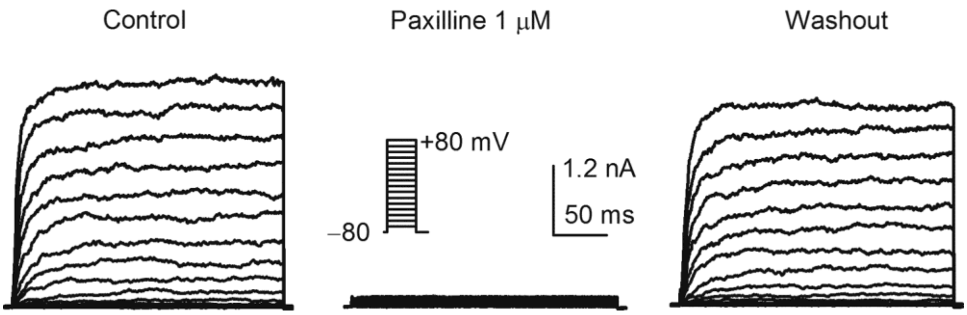

Figure S1. Membrane current in HEK 293 cells stably expressing hKCa1.1 (*KCNMA1*) and the auxiliary β1 subunit (*KCNMB1*) is inhibited by paxilline. Voltage-dependent hKCa1.1 current traces recorded in a representative cell with 300-ms voltage steps between -70 and +80 mV from a holding potential of -80 mV using a pipette solution contained 300 nM free Ca^{2+} in the absence and presence of 1 μM paxilline.

Figure S2

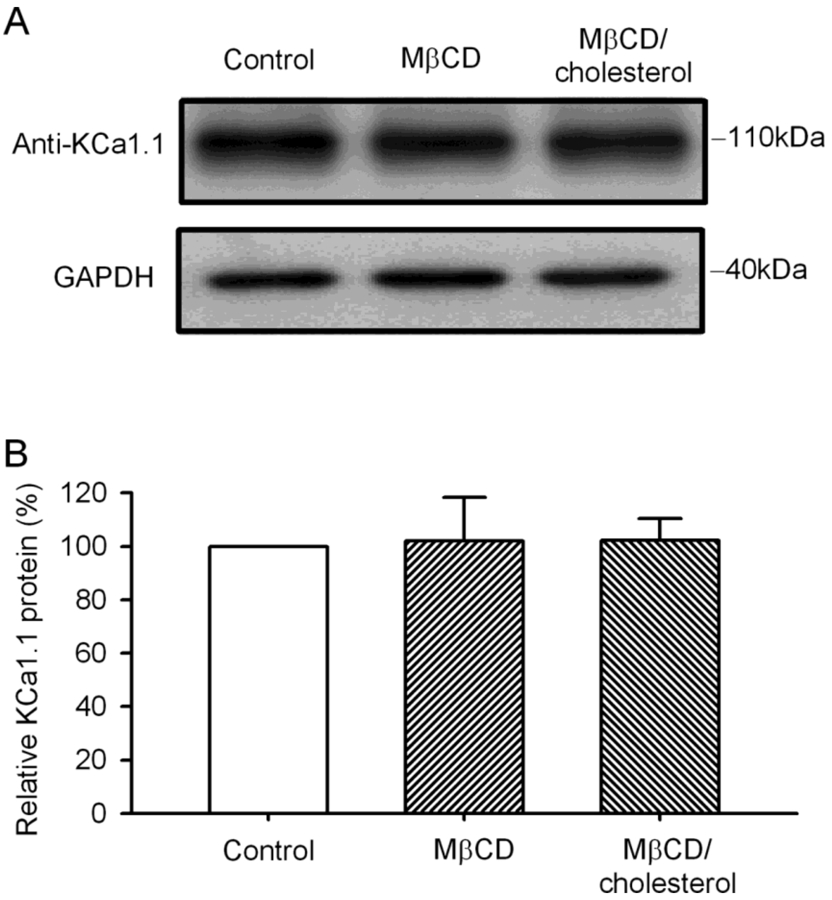

Figure S2. Cholesterol has no effect on KCa1.1 protein expression in hKCa1.1-HEK 293 cells without expressing auxiliary β1-subunits. **A.** Western immunoblots showing KCa1.1 protein in cells treated with cholesterol depletion (MβCD) or cholesterol enrichment (MβCD/cholesterol). **B.** Mean percentage values of KCa1.1 protein in cells with different treatments (n=4, P=NS vs. control).
